# Women of Worth: the impact of a cash plus intervention to enhance attendance and reduce sexual health risks for young women in Cape Town, South Africa

**DOI:** 10.1002/jia2.25938

**Published:** 2022-06-14

**Authors:** Tracey Naledi, Francesca Little, Carey Pike, Harley Edwards, Dante Robbertze, Colleen Wagner, Leslie London, Linda‐Gail Bekker

**Affiliations:** ^1^ Desmond Tutu HIV Centre, UCT Health Sciences Faculty University of Cape Town Cape Town South Africa; ^2^ Division of Public Health Medicine University of Cape Town Cape Town South Africa; ^3^ Department of Statistical Sciences University of Cape Town Cape Town South Africa

**Keywords:** conditional cash transfer, emerging adult women, HIV/AIDS, combination HIV interventions, young woman, youth

## Abstract

**Introduction:**

Conditional cash transfers (CTs) augmented with other interventions are promising interventions for reducing HIV risk in adolescent girls and young women.

**Methods:**

A multi‐phase, quasi‐experimental study assessed the impact of a CT (ZAR300; $22) conditional on attending a skills building intervention, Women of Worth (WoW), designed to improve sexual and reproductive health (SRH) outcomes in Cape Town, South Africa from May 2017 to December 2019. The intervention entailed 12 sessions with encouragement to attend adolescent and youth‐friendly health services. Women aged 19–24 years were randomized 1:1 to receive the intervention with a CT (“cash + care” or C+C) or without a CT (“care”). The study included a pilot phase followed by a post‐modification phase with improved uptake and retention without changing programme content or CT. Self‐reported HIV prevalence and SRH/HIV vulnerability were assessed via a self‐administered questionnaire at baseline, after 11 sessions, and 6–30 months’ post‐intervention for a subset. Mixed effect logistic regression models were fitted to estimate within‐subject changes in outcomes.

**Results:**

Of 5116 participants, 904 (452 participants per arm) were in the pilot and 4212 (2039 “care” participants and 2173 “C+C” participants) were in the post modified phase. There were 1867 (85.9%) and 135 (6,6%) participants in the “C+C” group and the “Care,” respectively, that were WoW completers (≥ 11 sessions/retention). During the pilot phase, 194 (42.9%) and 18 (4.0%) participants in “C+C” and the “care” groups were retained. Receiving a CT sustained participation nearly 60‐fold (OR 60.37; 95% CI: 17.32; 210.50, *p* <0.001). Three‐hundred and thirty women were followed for a median of 15.0 months [IQR: 13.3; 17.8] to assess the durability of impact. Self‐reported new employment status increased more than three‐fold (*p* <0.001) at WoW completion and was sustained to the longer time point. Intimate partner violence indicators were reduced immediately after WoW, but this was not durable.

**Conclusions:**

Participants receiving CT had sustained participation in an SRH/HIV prevention skills building with improvement in employment and some SRH outcomes. Layered, “young woman centred” programmes to address HIV and SRH risk in young women may be enhanced with CT.

## 1 INTRODUCTION

Despite recent gains in HIV control in Eastern and Southern Africa (ESA), progress for adolescent girls and young women (AGYW) (15–24 years old) remains unacceptably slow and off‐track to meet the UNAIDS 2030 goals [1]. The COVID‐19 pandemic amplified the disproportionate burden of biological, social, economic and structural risk factors that drive HIV vulnerability in AGYW [2, 3]. AGYW aspirations for a successful transition into adulthood are hampered by HIV vulnerability, low high school completion, unemployment, unintended pregnancies, poor sexual reproductive health (SRH)/HIV knowledge and persistent gender inequalities [4]. In South Africa (SA), half the population live below the poverty line of R 992 ($70) per month and a third are dependent on social grants [5, 6]. Half of those who are not in employment, education nor training are youth [6, 7]. The child support grant (CSG), a national unconditional cash transfer (CT) of R460 ($32) per month to children <18 years, has positively impacted childhood health outcomes and adolescent HIV vulnerability [8, 9]. Yet, the CSG ends at a time when increased financial susceptibility in young women can lead to transactional sex, inter‐generational, unequal sexual power relationships and increased HIV risk [10, 11].

The complexity of HIV vulnerability in young women has led to calls for multi‐deterministic approaches that combine behavioural and structural interventions delivered close to where they live [12, 13, 14]. These approaches aim to reduce individual, social and structural barriers to safe behaviours and enhanced access to services [15]. The effectiveness of CT alone on SRH/HIV outcomes in AGYW has been disappointing [16, 17] “Cash plus” interventions have been recommended as they augment CTs with life skills, behavioural interventions (BIs) and health systems strengthening that could promote protective SRH behaviours, as well as access to quality services [15, 16, 18, 19, 20]

A multi‐phase, quasi‐experimental study, Women of Worth (WoW), was conducted among 19‐ to 24‐year‐old women in Cape Town, SA as a component of an integrated, multi‐component, multi‐sector intervention for young people (aged 10–24 years) funded by the Global Fund for Malaria, HIV and TB [21]. We hypothesized that a CT conditional on the attendance of a modular SRH/HIV skills building programme and the simultaneous strengthening of adolescent and youth‐friendly services (AYFS) at public facilities would lead to reduction in risk factors and result in favourable SRH/HIV outcomes in this population. We aimed to determine the baseline characteristics of HIV vulnerability among young women; quantify retention in the programme with and without a conditional CT; and measure changes in the prevalence of HIV‐ and SRH‐related risk factors before and after exposure to the program.

## 2 METHODS

### 2.1 Study setting

The study was conducted across two sub‐districts in Cape Town, SA, with a combined population of approximately 1 million living in formal and informal urban settlements. This setting is characterized by high levels of HIV, crime, violence and socio‐economic deprivation [22, 23]. Most residents are isiXhosa or Afrikaans/English speaking. We implemented the intervention across 10 community venues within these sub‐districts using near‐peer community‐recruited facilitators.

### 2.2 Study population

Approximately 10,000 19‐ to 24‐year‐old women were recruited from the study area, regardless of HIV status. Inclusion criteria included residing in the study area and owning a mobile phone or having access to one. The latter facilitated CT payment. Recruitment was done through social media, word of mouth and community‐based organizations.

### 2.3 Intervention and study design

**Figure 1:**
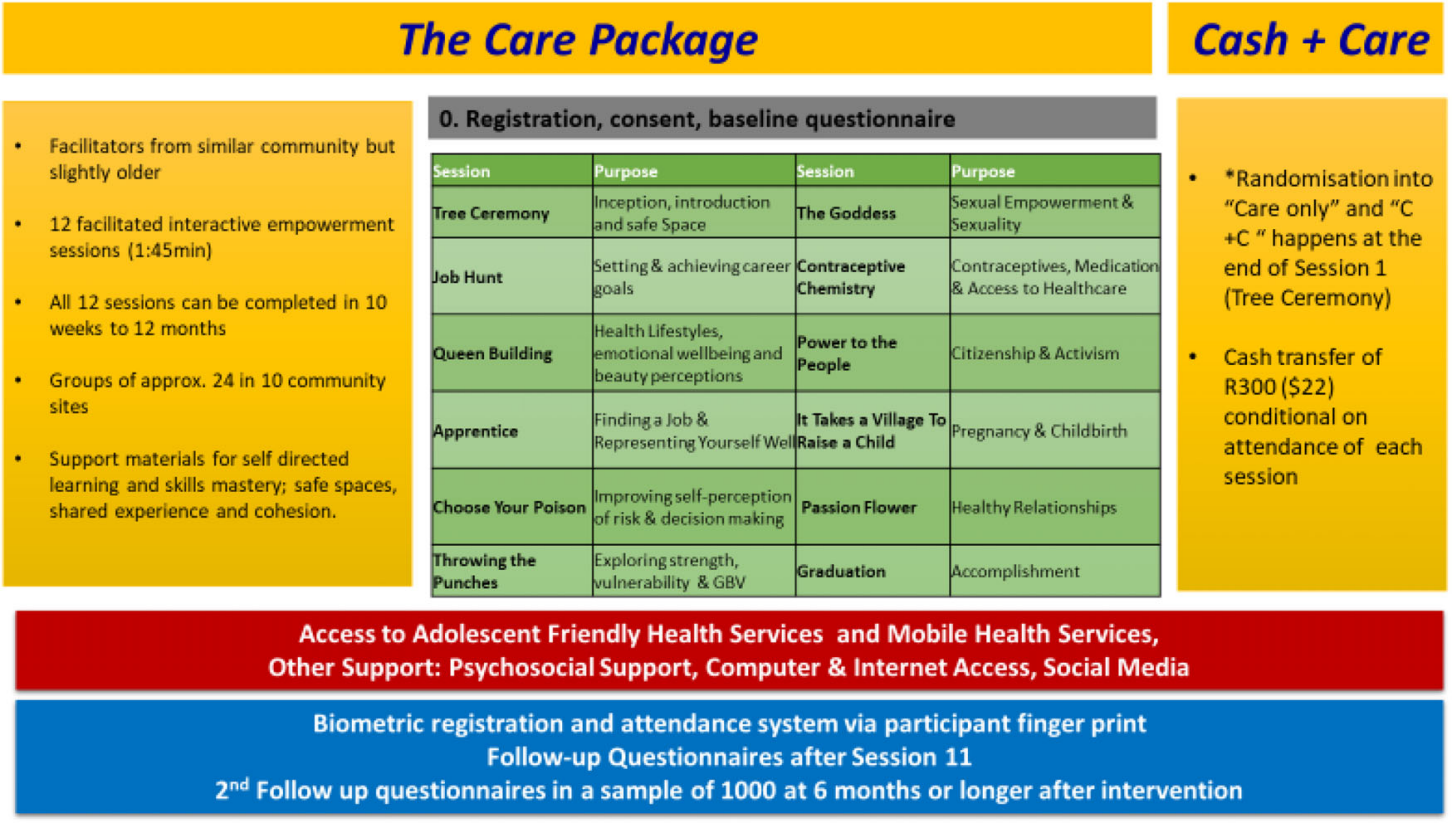
Women of Worth intervention schema.

The WoW program was adapted from a pilot intervention, GirlPower, implemented in SA and Malawi between 2016 and 2017 [24]. The facilitator‐led empowerment sessions addressed SRH/HIV knowledge, self‐esteem, healthy relationships, financial and health literacy (SRH/HIV, mental health and gender‐based violence [GBV]), job‐seeking skills and active citizenry [24]. Figure 1 shows the WoW “care” interventions that included (1) 12 facilitator‐led, group skills building sessions to address a range of SRH/HIV determinants, (2) support services, including psychosocial services and (3) fixed (government) and mobile (non‐governmental) YFHS with the promotion of HIV testing, contraception services, antiretroviral treatment and HIV pre‐exposure prophylaxis referral. The “care plus cash” (C+C) arm included this “care” package plus a CT of R300 ($22) paid after attendance at each session. Upon initiation, participants received a WoW T‐shirt, bag, water bottle and WoW materials. A light snack was offered after each session and graduation included a certificate and a meal.

We used a multi‐phase, quasi‐experimental mixed‐methods study design. Participants registered on‐site by self‐selection on a first‐come, first‐served basis by providing electronic informed consent and self‐administering an SRH/HIV risk assessment questionnaire. Questionnaires were conducted at baseline (registration) and following the 11th session.

In the initial randomized control trial (RCT) phase, approximately 5000 participants were randomized 1:1 to “care” arm versus “C+C” arm. After enrolling approximately 1000 participants in the pilot phase, low participant retention prompted a study pause between December 2017 and March 2018. The RE‐AIM approach was used to review the program and distil modifications solicited from staff and participant consultations shown in Table S1 [25]. Improvements applied to both study arms included implementation support and changing from fixed monthly sessions to weekly flexible attendance, such that sessions could be attended in any order prior to the 12th session (graduation). Programme content and CT remained unchanged. Facilitators underwent regular training, with unannounced fidelity assessments conducted on site. Participants already enrolled in the pilot phase continued in the modified programme. To determine the durability of effect, we invited participants who attended at least one session with post‐exposure time of between 6 and 30 months (total project months) to repeat the baseline questionnaire. We offered a R50 ($3,44) reimbursement on a first‐come, first‐served basis to a maximum of 1000 participants for this follow‐up visit. After the randomized phase, an open label phase enrolled approximately 5000 participants and only offered “care and cash.”

### 2.4 Data collection and management

Self‐administered questionnaire data and session attendance were collected on a digital tablet using a fingerprint‐enabled biometric information system. After registration, participants were assigned a unique participant identification number (PID). PIDs were randomly assigned to a study arm following a biometric check‐in and out at the first session. A message indicating arm assignment was automatically sent to participant's mobile phone. A digital voucher was sent after each session attendance via mobile phone to those randomized to the “C+C” arm. Participants in the “care” arm received a message of encouragement but no voucher.

### 2.5 Study outcomes and analytical methods

The primary outcome of self‐reported changes in HIV prevalence and secondary outcomes of self‐reported SRH/HIV‐related risk factors were measured after exposure to the skills building sessions during the RCT study phase. We compared baseline risk factors by study arm to assess whether randomization ensured comparable HIV vulnerability by testing differences between groups using chi‐square statistics with a *p* ≤ 0.05 threshold for statistical significance. As a measure of retention, WoW completers were defined as those who completed at least 11 sessions. We fitted logistic regression models with subject‐specific random for mixed effects, to estimate the intra‐individual change estimates of effect. We measured changes in self‐reported HIV, behavioural and structural SRH risks from baseline to (1) end of WoW and (2) at follow up (6–30 months post‐exposure) irrespective of WoW completion. Observations in the models included subjects at baseline and at these outer time points. Maximum‐likelihood estimation of the model parameters ensured unbiased estimates despite loss to follow up (LTFU) under the missing‐at‐random assumption. However, to mitigate potential bias due to unmeasured confounders or predictors of LTFU in the “care” arm, we analysed change in outcomes not disaggregated by study arm. Models included the number of visits attended to measure intervention dose. Models were adjusted for confounding variables that were (1) statistically different at baseline between the study arms and (2) baseline variables that were associated with LTFU at the end of WoW and at follow up (Table S2). All analyses were conducted using STATA 15 [26].

### 2.6 Ethics

The study design was discussed with community stakeholders and a local Community Advisory Board before protocol review. Due to concerns raised by community members, CTs were not conditional on uptake of health services but rather incentivized attendance at skills building sessions. Ethics approval was received from the University of Cape Town Human Research Ethics Committee (HREC) HREC 033/2017 and HREC 716/2018. The trial is registered with BMC Trials ISRCTN25016009 https://doi.org/10.1186/ISRCTN25016009.

## 3 RESULTS

### 3.1 Programme uptake

**Figure 2:**
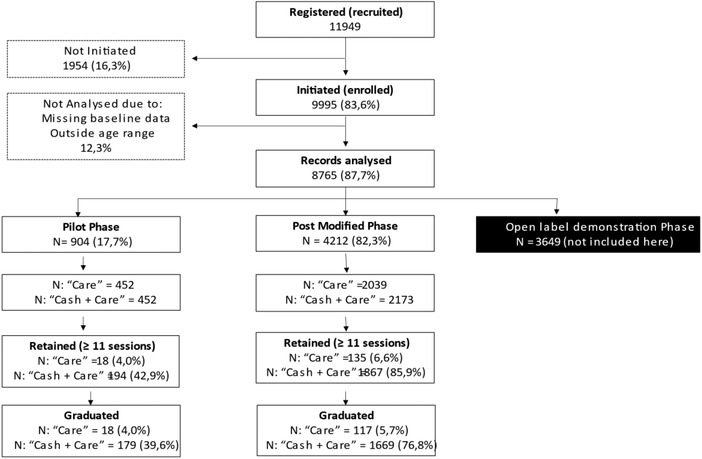
Consort diagram of recruitment, enrolment and retention in the WoW programme (RCT only).

Program participation is shown in Figure 2 with 11,494 participants registered and 83.6% (9995) successfully initiating the WoW program by attending at least one session. Complete records of 8765 (87.8%) initiators were usable and analysed. Only 904 and 4212 participants in the randomized pilot phase and post modification phases, respectively, are included here.

### 3.2 Baseline socio‐economic and structural vulnerabilities to HIV

Table 1 shows the comparable baseline characteristics of participants by study arm. Participants’ median age was 21.5 years IQR [20.2; 22.9]. Only 2391 (46.7%) completed high school and most 3724 (72.8%) were unemployed but actively seeking work. Half 2583 (50.5%) had no income with reported dependence on relatives and social grants and limited financial assistance from intimate partners. Despite high reported contraception usage, only 2068 (40.4%) reported condom use at last sex and nearly half (2361; 46.1%) the cohort reported a previous pregnancy and 1135 (22.2%) individuals received treatment for a sexually transmitted infection (STI) in the last 6 months. Although one third of individuals reported low HIV risk perception, almost all participants had undergone voluntary HIV testing in the last 6 months. Two‐thirds of those who were HIV positive reported to be on antiretroviral treatment, but viral load suppression was less than 20%. Gender‐based threats or violence by the current or last partner was experienced by one in five participants and 675 (13.2%) reported ever experiencing forced sex. Overall health facility satisfaction was relatively high at 3152 (61.6%). Most participants still reported dissatisfaction with long waiting times, travel distances and unfriendly staff. Because of programme modifications, the cohorts from the pilot and post modification were compared at baseline. The two cohorts were similar except the post modification cohort had a lower socio‐economic profile and lower reported HIV prevalence (5.1%) versus those in the pilot phase (8.2%).

**Table 1:** Baseline socio‐economic and structural vulnerabilities to HIV by study arm and study phase

	**Pilot (*N* = 904)**					**Post modification (*N*= 4212)**					**All phases randomized (*N* = 5116)**					**All phases, all arms (*N* = 5116)**						
	**Care only *N* = 452**		**C+C**	***N* = 452**	***p value***	**Care only *N* = 2039**		**C +C**	***N* = 2173**	***p value***	**Care only *N* = 2491**		**C+ C**	***N* = 2625**	***p value***	**Pilot**	***N* = 904**	**Post modification *N* = 4212**		**Total *N* = 5116**		***p* value**
*MeanAge [95% CI*	21.0	(18.6; 24.7)	21.0	(18,7; 24.7)		21.0	(18.6; 24.9)	21.0	(18.5; 24.9)		21.0	(18.6; 24.9)	21.0	(18.5; 24.9)		21.0	(18.6; 24.7)	21.0	(18.6; 24.7)	21	(18.5; 24.7)	
*Gender: Female*	444	98.2%	437	96.7%	*0.139*	2037	99.9%	2166	99.7%	*0.116*	2481	99.6%	2603	99.2%	*0.048*	881	97.5%	4203	99.8%	5084	99.4%	<0.001
*Language: isiXhosa*	343	75.9%	342	75.7%	*0.938*	1726	84.7%	1875	86.3%	*0.132*	2069	83.1%	2217	84.5%	*0.175*	685	75.8%	3601	85.5%	4286	83.8%	<0.001
**Socio‐economic determinant indicators**																						
*Completed high school*	215	47.6%	195	43.1%	*0.181*	949	46.5%	1032	47.5%	*0.537*	1164	46.7%	1227	46.7%	*0.992*	410	45.4%	1981	47.0%	2391	46.7%	0.359
*Unemployed but actively seeking*	346	76.6%	344	76.1%	*0.876*	1482	72.7%	1552	71.4%	*0.362*	1828	73.4%	1896	72.8%	*0.353*	690	76.3%	3034	72.0%	3724	72.8%	0.008
*No income*	186	41.2%	168	37.2%	*0.220*	1115	54.7%	1114	51.3%	*0.026*	1301	52.2%	1282	48.8%	*0.015*	354	39.2%	2229	52.5%	2583	50.5%	<0.001
*Financial assistance from social grant*	93	20.6%	124	27.4%	*0.016*	425	20.8%	490	22.6%	*0.180*	518	20.8%	614	23.4%	*0.025*	217	24.0%	915	21.7%	1132	22.1%	*0.134*
*Financial assistance from intimate partner*	17	3.8%	21	4.7%	*0.507*	88	4.3%	93	4.3%	*0.954*	105	4.2%	114	4.3%	*0.822*	38	4.2%	219	4.3%	257	5.0%	*0.900*
*Relationship status: cohabiting/married*	53	11.7%	45	10.0%	*0.392*	117	5.7%	142	6.5%	*0.282*	170	6.8%	187	7.1%	*0.675*	98	10.8%	259	6.2%	357	7.0%	<0.001
**Psychosocial wellbeing determinants indicator**																						
*Happy/content/optimistic*	337	74.6%	332	73.5%	*0.705*	1228	60.2%	1238	57.0%	*0.032*	1565	62.8%	1570	59.8%	*0.027*	669	74.0%	2466	58.5%	3135	61.3%	<0.001
*Family very supportive*	321	71.0%	317	70.1%	*0.770*	968	47.5%	1015	46.7%	*0.619*	1289	51.8%	1332	50.8%	*0.473*	638	70.6%	1983	47.1%	2621	51.2%	<0.001
*Alc drink 5 or more days per week*	38	8.4%	36	8.0%	*0.808*	158	7.8%	182	8.4%	*0.456*	196	7.9%	218	8.3%	*0.567*	74	8.2%	340	8.1%	414	8.1%	0.909
*Using drugs in the last 3 months*	22	4.9%	20	4.4%	*0.752*	103	5.1%	125	5.8%	*0.315*	125	5.0%	145	5.3%	*0.419*	42	4.7%	228	5.4%	270	5.3%	0.349
**Sexual Reproductive Health (SRH) behavioural Indicators**																						
*Ever tested for HIV*	404	89.4%	421	93.1%	*0.045*	1879	92.2%	2023	93.1%	*0.241*	2283	91.7%	2444	93.1%	*0.050*	825	91.3%	3902	92.6%	4727	92.4%	0.156
*HIV test in the last 6 months (378 missing records)*	302/405	74.6%	331/422	78.4%	*0.189*	1549/1884	82.2%	1661/2027	81.9%	*0.823*	1851/2289	80.9%	1992/3843	81.3%	*0.677*	633/827	76.5%	3210/3911	82.1%	3843	81.1%	<0.001
*Condom use at last sex*	164	36.3%	162	35.8%	*0.890*	845	41.4%	897	41.3%	*0.915*	1009	50.5%	1059	40.3%	*0.905*	326	36.1%	1742	41.4%	2068	40.4%	0.003
*STI Rx in the last 6 months*	90	19.9%	98	21.7%	*0.512*	478	23.4%	469	21.6%	*0.148*	568	22.8%	567	21.6%	*0.301*	188	20.8%	947	22.5%	1135	22.2%	0.268
*High HIV risk perception*	141	31.2%	169	37.4%	*0.050*	724	35.5%	721	34.3%	*0.112*	865	34.7%	890	33.9%	*0.537*	310	34.3%	1445	34.3%	1755	34.3%	0.993
*Ever used contraception*	313	69.3%	316	69.9%	*0.828*	1447	71.0%	1563	71.9%	*0.490*	1760	70.7%	1879	71.6%	*0.465*	629	69.6%	3010	71.5%	3639	71.1%	0.257
*On contraception currently*:	281	62.2%	290	64.2%	*0.535*	1313	64.4%	1449	66.7%	*0.118*	1594	64.0%	1739	66.3%	*0.09*	571	63.2%	2762	65.2%	3333	65.1%	0.168
*Ever pregnant*	216	47.8%	217	48.0%	*0.947*	927	45.5%	1001	46.1%	*0.695*	1143	45.9%	1218	46.4%	*0.712*	433	47.9%	1928	45.2%	2361	46.1%	0.245
**HIV outcome indicators**																						
*HIV positive*	37	8.2%	37	8.2%	*1.000*	102	5.0%	114	5.3%	*0.720*	139	5.6%	151	5.8%	*0.790*	74	8.2%	216	5.3%	290	5.7%	<0.001
*HIV positive on ART*	21/37	56.8%	22/37	59.5%	*0.814*	69/102	67.7%	75/114	65.8%	*0.772*	90/139	64.8%	97/151	64.5%	*0.928*	43/74	58.1%	144/216	66.7%	187	64.5%	0.184
*HIV‐positive VL suppressed*	7/37	18.9%	4/37	10.8%	*0.327*	21/102	20.6%	20/114	17.5%	*0.569*	28/139	20.1%	24/151	15.9%	*0.346*	11/74	14.9%	41/216	19.0%	52	17.9%	0.426
**Structural determinants Indicators**																						
*GBV threat*	65	14.4%	71	15.7%	*0.577*	426	20.9%	476	21.9%	*0.423*	491	19.7%	547	20.8%	*0.316*	136	15.0%	902	21.4%	1038	20.3%	<0.001
*Forced sex ever*	36	8.0%	46	10.2%	*0.247*	278	13.6%	315	14.5%	*0.422*	314	12.6%	361	13.8%	*0.226*	82	9.1%	593	14.1%	675	13.2%	<0.001
*Transactional sex ever*	29	6.4%	19	4.2%	*0.138*	359	17.6%	353	16.2%	*0.239*	388	15.6%	372	14.2%	*0.158*	48	5.3%	712	16.9%	760	14.9%	<0.002
*Overall health facility satisfaction*	299	66.2%	303	67.0%	*0.778*	1246	61.1%	1304	60.0%	*0.466*	1645	62.0%	1607	61.2%	*0.554*	602	66.6%	2550	60.5%	3152	61.6%	0.001
*Dissatisfaction: waiting time*	276	61.1%	261	57.7%	*0.770*	1129	55.4%	1147	52.8%	*0.305*	1405	56.4%	1408	53.6%	*0.185*	537	59.4%	2276	54.0%	2813	55.0%	
*Dissatisfaction: unfriendly staff*	53	11.7%	57	12.6%		339	16.6%	390	18.0%		392	15.7%	447	17.0%		110	12.2%	729	17.3%	839	16.4%	
*Dissatisfaction: travel*	83	18.4%	88	19.5%		349	17.1%	374	17.2%		432	17.3%	462	17.6%		171	18.9%	723	17.2%	894	17.5%	<0.001

### 3.3 Program retention by arm and study phase

**Figure 3:**
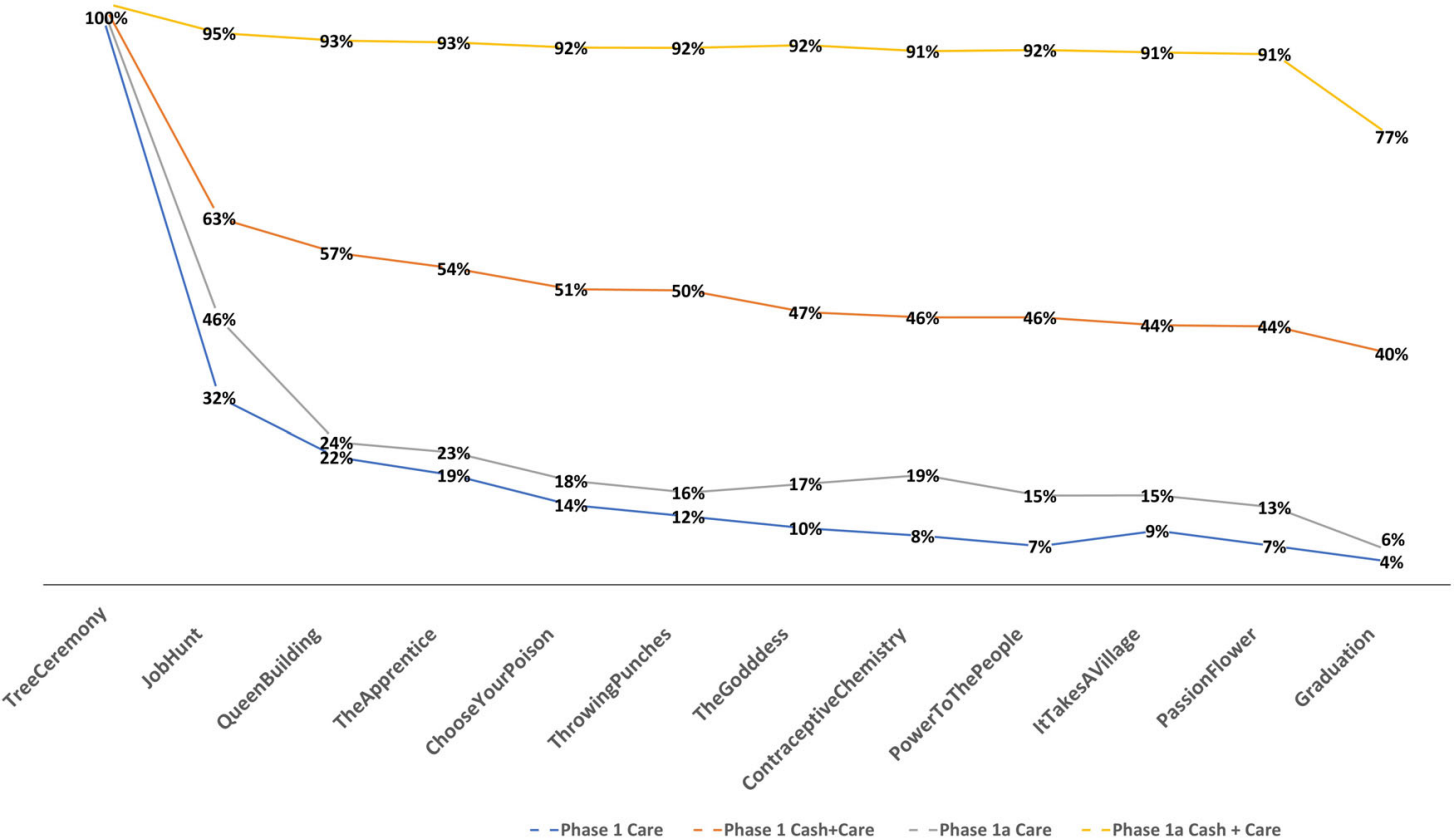
Retention in the Women of Worth programme by study arm and study phase.

Two thousand and two hundred and fourteen (43.3%) completed the programme, with significant differences in retention between study arms and phases (Figure 2). Of “C+C” participants enrolled in the modified programme, 1867 (85.9%) were WoW completers and 1669 (76.8%) continued to graduation. Of those enrolled in the pilot phase, only 194 (42.9%) participants in the “C+C” arm were WoW completers and 179 (39.6%) graduated. Overall, 1848 (78.5%) participants in the “C+C” arm were retained. In contrast, participants randomized to “care” in all study phases were poorly retained with a steep reduction at the end of the first session after randomization was applied (Figure 3). Following modification, in the “care” arm, only 135 (6.6%) participants were WoW completers and 117 (5.7%) graduated. Similarly, in the pilot phase “care” arm, 18 (4.0%) participants completed 11 sessions and 11 graduated. Compared with “care” arm participants, “C+C” arm participants were 60 times more likely to be WoW completers (OR 60.37; 95% CI: 17.32; 210.50, *p* <0.001). We saw a 23‐fold improvement in retention of participants in the post modification phase (OR 22.91; 95% CI: 1.07; 516.39; *p* = 0.049) versus the pilot phase. Baseline variables associated with LTFU (Table S2a and S2b) and those that had a statistically significant association with LTFU at the end of WoW (completers vs. non‐completers) and at median 15 months (followed up vs. not followed up) were used to adjust the models discussed below.

### 3.4 SRH outcomes by arm

Of the 2214 “WoW completers,” 1149 (51.9%) completed questionnaires after 11 sessions: 1005 “C+C” and 144 “care.” Overall, the “C+C” and “care” groups completed the programme in a similar duration of median 15 weeks (IQR: 12 weeks) and 14 weeks (IQR: 12 weeks), respectively. The post modification phase had a similar completion time between arms of median 14 weeks with a shorter IQR of 7 weeks. Participants who enrolled during the pilot phase had a longer completion time of median 46 weeks (IQR: 33 weeks).

Adjusted changes in self‐reported SRH behavioural and structural risk factors immediately after WoW with both study arms combined are shown in Table 2. Compared to baseline, changes in self‐reported structural risk factors at the end of WoW were consistent. The odds of self‐reporting current employment status increased more than three‐fold (*p* <0.001) and self‐reporting facility satisfaction increased 45% (*p* <0.001). The odds of self‐reporting GBV threat, forced sex and transactional sex were reduced by 47% (*p* <0.001), 63% (*p* <0. 001) and 50% (*p* <0.001), respectively.

**Table 2:** Impact of WoW on intra‐subject self‐reported behavioural and structural risk factors for HIV vulnerability in women who completed the WoW program and those followed up at median 15 months

At the end of WoW (*N* = 1149)^a,b^	OR	95% CI		*p* Value
HIV test in the last 6 months	0.25	0.20	0.31	<0.001
Condom use at last sex	0.49	0.40	0.60	<0.001
High HIV risk perception	0.05	0.03	0.08	<0.001
Current contraception	1.62	1.29	2.03	<0.001
Treated STI in the last 6 months	1.50	1.21	1.85	<0.001
GBV threat	0.53	0.41	0.69	<0.001
Forced sex	0.37	0.27	0.52	<0.001
Transactional sex	0.50	0.37	0.66	<0.001
Employed	3.34	2.22	5.04	<0.001
Facility satisfaction	1.45	1.20	1.75	<0.001

At follow up (*N* = 330)^c,d^	OR	95% CI		*p* value
HIV test in the last 6 months	0.70	0.12	4.15	0.696
Condom use at last sex	0.91	0.98	1.02	0.593
High HIV risk perception	0.89	0.71	1.13	0.350
Current contraception	1.00	0.77	1.29	0.973
Treated STI in the last 6 months	1.09	0.99	1.34	0.432
GBV threat	0.99	0.76	1.30	0.964
Forced sex	0.75	0.50	1.11	0.152
Transactional sex	0.83	0.63	1.10	0.200
Employed	2.47	1.69	3.59	< 0.001
Facility satisfaction	0.90	0.72	1.12	0.373

^a^Adjusted for session attendance, study phase, language:isiXhosa, happiness and family support.

^b^Observations include responses at baseline (*N* = 5116) and at the end of WoW (*N* = 1149).

^c^Adjusted for session attendance, study phase, language:isiXhosa, high school completion, family support, HV testing in the last 6 months and transactional sex.

^d^Observations include responses at baseline (*N* = 5116) and at median 15 months (*N* = 330).

The changes on SRH behavioural risk factors were mixed. The odds of self‐reporting uptake of contraception and STI treatment increased by 62% (*p* < 0.001) and 50% (*p*< 0.001), respectively, at the end of WoW compared to baseline. However, comparing the same period, the odds of self‐reporting HIV testing in the last 6 months, condom use at last sex and recognition of HIV risk perception were reduced by 75% (*p* <0.001), 51% (*p*< 0.001) and 95% (*p* < 0.001), respectively. In addition to the 290 participants who self‐reported HIV positivity at baseline, 42 participants at the end of WoW and seven participants at follow up self‐reported HIV positivity for the first time. These numbers were insufficient to model and assess new HIV infection.

### 3.5 Durability of impact of the WoW program

There were 330 participants (132 “care” and 198 “C+C”) of the 1000 participants invited to test for durability of effect and the dose response of the skills building sessions who were in the randomized phases of the study. They had follow‐up time of median 15.0 months [IQR: 13.3; 17.8] post‐intervention. In this group, 206 (62.4%) attended ≥9 sessions and 102 (30.9%) ≤ 3 sessions. At this time point, the odds of self‐reported current employment status showed durability and were increased 2.5‐fold (*p* <0.001) compared to baseline (Table 2). There was no discernible dose response.

## 4 DISCUSSION

Receipt of a modest CT increased young women's sustained participation in the WoW SRH skills building programme by 60‐fold. Finding and holding participant attention in such programs, especially in the context of competing priorities, is difficult. Young women who have recently completed secondary school are highly transient and difficult to retain, as illustrated by the limited number who could be located for long‐term follow up. Yet, SRH impact depends on sufficient session exposure, making session attendance critical [26, 27, 28]. Mechanistically, conditional CT may provide a means to capture and hold participants’ attention and so support sustained engagement and increased exposure to session content [16]. The benefit of the WoW program alone remains underdetermined, as attendance in the “care” only arm was very low. However, after controlling for cofounders in those retained, current employment status increased more than three‐fold immediately after WoW, and this was durable post‐intervention.

Baseline characteristics demonstrated low socio‐economic profiles with high rates of unemployment and HIV prevalence and risk behaviours, similar to population characteristics in the study areas [29]. Youth constitute a large proportion of the unemployed in Africa, making them harder to reach and retain in interventions due to competing demands of job seeking [30, 31]. The incongruent, poor HIV risk perception, is a significant predictor for the adoption of health‐promoting behaviour. In our participants this may result from HIV being deprioritized in their overall context, as there is evidence that poverty‐related stressors, such as unemployment, low education and community violence, may override stressors or risk perceptions related to HIV/AIDS [32]. The high LTFU in the “care” group suggests that rewarding attendance, even modestly, may be necessary to “nudge” individuals to engage. This maximizes their exposure to developmental interventions, which, when combined with structural and BIs, have potential to reduce HIV vulnerability [16].

Curbing unemployment addresses a structural determinant of HIV vulnerability in AGYW and a sustainable development priority [32, 33]. Unemployment at baseline was high in all groups even in those LTFU. This makes the generalizability of the finding of increased employment to poor, urban, out‐of‐school, young women in Cape Town plausible. In SA, unemployment increased in young people of a similar socio‐economic profile during the study period, further suggesting credibility of our findings [34]. However, as employment was self‐reported, social desirability and other unmeasured confounders may have biased these results. However, as two of the 12 WoW sessions addressed job seeking, WoW may have directly improved their skills and competence. Participants were provided with computer and internet access as well as assistance in CV development and job applications. That the effect of these interventions was durable for more than a year after exposure is encouraging albeit based on a small sample size.

Our findings on behavioural risk factors were mixed. WoW showed promise in the short term by increasing individuals’ uptake of contraception and STI treatment, while condom use and recognition of HIV risk remained unchanged. SRH behaviour change in AGYW is complex, and patriarchal power dynamics frame perceptions, norms and SRH behaviours and influence HIV vulnerability [35, 36]. CT studies in AGYW in SA and Tanzania have shown reductions in risky sexual behaviours by using financial resources to improve bargaining power in sexual relationships and reducing the number of sexual partners [37, 38]. A review of CT effectiveness on GBV found mixed results, although augmentation with “gender transformative” and skills building interventions improved outcomes [39, 40]. This corroborates with WoW program results, which included a GBV component.

Evidence for the effectiveness of financial incentives to impact health behaviours, such as medication adherence, linkage and retention in care, has so far shown mild, unstained outcomes [41, 42, 43]. CTs augmented with human development and “supply‐side” interventions may be more effective as they increase individual and institutional resources and capacities for demand creation and service quality [44]. Even though no incentives were given in WoW to directly motivate services access; the attention to overall quality improvement and promotion of AYFS health services during sessions may have partially addressed “supply issues” that could have influenced contraception and STI treatment uptake [45, 46]. Increased facility satisfaction suggested that AFYS may have impacted service quality, although this effect was not durable showing the difficulty of sustaining health service improvements.

CTs with augmented HIV interventions in African AGYW have shown potential to lower HSV‐2, reduce HIV incidence to various degrees, reduce transactional sex and reduce HIV risk behaviours [24, 47, 48, 49, 50, 51]. Within these programmes, CTs have been strongly associated with attendance and/or adherence with the study intervention [24, 50, 52]. WoW demonstrated the value of a “young woman centred” that uses a CT to ensure program participation while addressing transitional AGYW needs. These findings add to the emerging evidence in support of multi‐component “layered” interventions to reduce HIV vulnerability in AGYW in Africa [24, 27, 50]. The durability of some WoW outcomes 15 months post intervention suggests an effect in the absence of any further reward.

The appropriate size of the CT is determined by the objectives of the intervention; however, the World Bank has found that CT in ESA needs to provide >20% of household consumption to have a significant impact on health and development [53]. Baird and colleagues did not show improved health outcomes with small increases in CT to AGYW in Malawi [54]. The CT given to WoW participants was about 33% less than the value of the national CSG for 1 year and 30% of the national poverty line [5]. This suggests that modest CTs are more likely a “nudge” than a livelihood. In the context of HIV prevention, this intervention could be regarded as high cost; however, the cost‐effectiveness, which includes a sustained increase in employment that could impact beyond HIV, is undetermined.

This study used self‐reported measures, which may provide unreliable indications of actual behaviours due to recall and social desirability biases. Participants could report different HIV status results at each follow‐up visit potentially biasing the direction of the results. HIV‐positive results were, however, retained for all visits regardless of subsequent responses. Mitigation of the LTFU limitations by linking study records with provincial HIV records was unsuccessful. HIV status reliability in this and future programmes could be strengthened by the inclusion of obligatory HIV/STI testing pre and post intervention to validate self‐reports, though this limits voluntary uptake of testing. The lack of sustained participation in the “care” arm was the greatest confounder and impacted study power and effect sizes. Lack of durability data is a limitation in CT studies, thus, our follow‐up data provide important insights [50, 55, 56]. However, caution should be exercised due to the small sample size. Qualitative studies to gain participant insights on this and the role of the cash incentives in their lives are underway to inform potential effect pathways.

## 5 CONCLUSIONS

A modest CT conditional on attendance of a modular SRH/HIV prevention skills building programme was necessary to incentivize and sustain participation of urban out of school young women in SA. Sustained participation resulted in increased current employment status and moderate improvement of some SRH aspects short term. Uptake and attendance in layered, “young woman centred” developmental programmes to address outcomes of HIV and SRH in young women may be enhanced with modest CT.

## COMPETING INTERESTS

There are no competing interests.

## AUTHORS’ CONTRIBUTIONS

TN conceived of the presented idea with LGB, performed fidelity testing and interviews, and undertook the data management, analysis and wrote the manuscript. FL provided technical guidance and review of the statistical analysis. CP helped develop the skills building manual and program, developed materials and reviewed the manuscript. HE managed the research team and reviewed the manuscript. DR trained facilitators, performed fidelity testing and interviews, and reviewed the manuscript. CW managed the research team and reviewed the manuscript. LL reviewed data analysis and interpretation of the manuscript. LGB conceived of the study design, helped to develop the skills building manual and program and materials, supervised the entire research process, including data analysis and interpretation, and reviewed the manuscript.

## FUNDING

The Global Fund for HIV, TB and Malaria funded program costs via the Western Cape Department of Health, The Desmond & Leah Tutu Foundation and the Discovery Foundation fund TN's PhD.

## DISCLAIMER

Nothing to report.

## Supporting information


**Table S1**: Intervention optimization defined by RE‐AIM Criteria

**Table S2a**: Baseline characteristics that are different in WoW completers compared to non‐completers

**Table S2b**: Baseline characteristics that are different in those at follow‐up compared to those not at follow‐up

## Data Availability

The authors confirm that the data supporting the findings of this study are available.
