# Supplementary material for: Women of Worth: the impact of a cash plus intervention to enhance attendance and reduce sexual health risks for young women in Cape Town, South Africa
Source: J Int AIDS Soc. 2022 Jun 14;25(6):e25938. doi: 10.1002/jia2.25938 (PMC9196891; doi:10.1002/jia2.25938)
Supplement: Supplementary file 1 — Table S1: Intervention optimization defined by RE‐AIM Criteria [file JIA2-25-e25938-s003.docx]

**Supplementary Table 1: Intervention optimisation defined by RE-AIM Criteria**

| RE-AIM criteria | WoW Pilot Phase | WoW Post Modification Phase |
| --- | --- | --- |
| **Reach** | Central registration office | Decentralised recruitment to each of the community venues |
|  | Referrals from community outreach teams working door to door, local NGO’s, health facilities and local media. | Word-of-mouth; and an incentivised recruitment strategy (WoW Sisters) which was however poorly promoted and implemented with low uptake. |
| **Effectiveness** | Intervention effectiveness Measures unchanged | Intervention effectiveness Measures unchanged |
| **Adoption** | Attendance schedule for the empowerment sessions only monthly | Attendance schedule for the empowerment sessions at least weekly or monthly if so wishes |
|  | Participants attend only at site of enrolment as part of a cohort. Session schedule determined by facilitator on site | Fixed venue session timetables and flexibility to attend sessions in any order and at any one of the community sites.  The first two and/or the last two sessions could be attended in the same week |
|  | Inter-session self-learning activities a requirement | Inter-session self-learning activities optional as participants perceived this as “homework”. |
| **Implementation** | Roving research teams responsible for multiple sites | A dedicated team of a facilitator and an administrator per site. The administrators managed amongst others the biometric system, which meant that the facilitators could focus exclusively on session delivery and rapport building with the participants. |
|  | Strict adherence to training manual | Facilitators allowed to tailor the sessions according to the issues that seemed to resonate with participants however we expected them to deliver minimum key messages per session and this was monitored by unannounced visits for fidelity testing |
|  | Paper based register at health facilities | Electronic bar-coding system (QR Codes) to track linkage to care of WoW participants to health services and incentive recruitment programme  Active promotion of health and psychosocial services and referral to health facility |
|  | In kind donation of the use of community venues which was unreliable and had poor safety and security | Poor safety and security and lack of reliability in the community venues was solved by renting selected and improved venues with enhanced availability, reliability, and security. |
| **Maintenance** | Project Manager providing support | Implementation monitored through the unannounced fidelity testing visits. |
|  | Once off training at the beginning of the programme | Training and enhanced support for facilitators (Dedicated trainer recruited, researchers). Ongoing training, technical updates, mentoring and on-site self-development coaching. |
|  | Ad hoc training, on site mentoring with limited capacity | Central weekly site reviews of uptake and retention data presented in full WoW operational meeting for healthy site competition |
