# Supplementary material for: Women of Worth: the impact of a cash plus intervention to enhance attendance and reduce sexual health risks for young women in Cape Town, South Africa
Source: J Int AIDS Soc. 2022 Jun 14;25(6):e25938. doi: 10.1002/jia2.25938 (PMC9196891; doi:10.1002/jia2.25938)
Supplement: Supplementary file 2 — Table S2a: Baseline characteristics that are different in WoW completers compared to non‐completers [file JIA2-25-e25938-s002.docx]

**Supplementary Table 2a:** **Baseline characteristics that are different in WoW completers compared to non-completers**

| At baseline  N = 5116 | WoW Completers  N = 2214 | % | No Completers  N = 2902 | % | P value |
| --- | --- | --- | --- | --- | --- |
| Study Phase (post modification) | 2002 | 90.4 | 2210 | 76.2 | ***<0.001*** |
| Language: isiXhosa | 1893 | 85.5 | 2393 | 82.5 | ***0.003*** |
| Completed High School | 1059 | 52.2 | 1332 | 45.9 | 0.170 |
| No Income | 1120 | 50.6 | 1463 | 50.4 | 0.902 |
| Cohabit OR Married | 154 | 7.0 | 203 | 7.0 | 0.956 |
| Happiness | 1312 | 59.3 | 1823 | 62.8 | ***0.010*** |
| Family Supportive | 1092 | 49.3 | 1529 | 52.7 | ***0.017*** |
| Binge Drinking | 186 | 8.4 | 228 | 7.9 | 0.479 |
| Drugs in Last 3 months | 119 | 5.4 | 151 | 5.2 | 0.786 |
| Current Contraception | 1475 | 66.6 | 1858 | 64.0 | ***0.053*** |
| HIV test in last 6 months | 1698/2071 | 82.0 | 2145/2667 | 80.4 | 0.173 |
| Condom use at last sex | 920 | 41.6 | 1148 | 39.6 | 0.150 |
| High HIV risk perception | 734 | 33.2 | 1021 | 35.2 | 0.130 |
| Treated STI in last 6 months | 486 | 22.0 | 649 | 22.4 | 0.725 |
| HIV positive | 123 | 5.6 | 167 | 5.8 | 0.760 |
| GBV threat | 469 | 21.2 | 569 | 16.6 | 0.165 |
| Forced sex | 314 | 14.2 | 361 | 12.4 | 0.068 |
| Transactional sex | 335 | 15.1 | 425 | 14.7 | 0.628 |
| Employed | 17 | 0.77 | 33 | 1.14 | 0.405 |
| Facility Satisfaction | 1363 | 61.6 | 1789 | 61.6 | 0.951 |
