# Supplementary material for: Women of Worth: the impact of a cash plus intervention to enhance attendance and reduce sexual health risks for young women in Cape Town, South Africa
Source: J Int AIDS Soc. 2022 Jun 14;25(6):e25938. doi: 10.1002/jia2.25938 (PMC9196891; doi:10.1002/jia2.25938)
Supplement: Supplementary file 3 — Table S2b: Baseline characteristics that are different in those at follow‐up compared to those not at follow‐up [file JIA2-25-e25938-s001.docx]

**Supplementary Table 2b: Baseline characteristics that are different in those at follow-up compared to those not at follow-up.**

| At baseline  N = 5116 | Follow up  N = 330 | % | No Follow up  N = 4779 | % | P value |
| --- | --- | --- | --- | --- | --- |
| Study Phase (post modification) | 259/330 | 78.5 | 3953/4786 | 82.6 | ***0.058*** |
| Language: isiXhosa | 316/330 | 95.8 | 3970/4786 | 83.0 | ***<0.001*** |
| Completed High School | 195/330 | 59.1 | 2196/4786 | 45.9 | ***<0.001*** |
| No Income | 179/330 | 54.2 | 2404/4786 | 550.2 | 0.159 |
| Cohabit OR Married | 20/330 | 6.1 | 337/4786 | 7.0 | 0.499 |
| Happiness | 215/330 | 65.2 | 2920 | 61.0 | 0.135 |
| Family Supportive | 186/330 | 56.4 | 2435/4786 | 50.9 | ***0.054*** |
| Binge Drinking | 24/330 | 7.3 | 390/4786 | 8.2 | 0.572 |
| Drugs in Last 3 months | 6/330 | 1.82 | 264/4786 | 5.52 | ***0.004*** |
| Current Contraception | 226/330 | 68.5 | 3107/4786 | 64.9 | 0.188 |
| HIV test in last 6 months | 273/316 | 86.4 | 3570/4422 | 80.7 | ***0.013*** |
| Condom use at last sex | 143/330 | 43.3 | 1925/4786 | 40.2 | 0.265 |
| High HIV risk perception | 98/330 | 29.7 | 1657/4786 | 34.6 | ***0.068*** |
| Treated STI in last 6 months | 74/330 | 22.4 | 1061/4786 | 22.2 | 0.914 |
| HIV positive | 18/330 | 5.5 | 272/4786 | 5.7 | 0.862 |
| GBV threat | 67/330 | 20.3 | 971/4786 | 20.3 | 0.995 |
| Forced sex | 37/330 | 11.2 | 638/4786 | 13.3 | 0.271 |
| Transactional sex | 36/330 | 10.9 | 724/4786 | 15.1 | ***0.037*** |
| Employed | 11/330 | 3.33 | 148/4786 | 3.09 | 0.807 |
| Facility Satisfaction | 206/330 | 62.42 | 2946/4786 | 61.6 | 0.753 |
